# Durability of Immune Response After COVID-19 Booster Vaccination and Association With COVID-19 Omicron Infection

**DOI:** 10.1001/jamanetworkopen.2022.31778

**Published:** 2022-09-15

**Authors:** Mayan Gilboa, Gili Regev-Yochay, Michal Mandelboim, Victoria Indenbaum, Keren Asraf, Ronen Fluss, Sharon Amit, Ella Mendelson, Ram Doolman, Arnon Afek, Laurence S. Freedman, Yitshak Kreiss, Yaniv Lustig

**Affiliations:** 1 Infection Prevention and Control Unit, Sheba Medical Center, Tel Hashomer, Ramat Gan, Israel; 2 Sackler School of Medicine, Tel-Aviv University, Tel Aviv, Israel; 3 Central Virology Laboratory, Public Health Services, Ministry of Health, Tel-Hashomer, Ramat Gan, Israel; 4 Dworman Automated-Mega Laboratory, Sheba Medical Center, Tel-Hashomer, Ramat-Gan, Israel; 5 Biostatistics and Biomathematics Unit, Gertner Institute of Epidemiology and Health Policy Research, Sheba Medical Center, Tel Hashomer, Israel; 6 Clinical Microbiology, Sheba Medical Center, Tel Hashomer, Ramat Gan, Israel; 7 General Management, Sheba Medical Center, Tel Hashomer, Ramat Gan, Israel

## Abstract

**Importance:**

The BNT162b2 two-dose vaccine (BioNTech/Pfizer) has high effectiveness that wanes within several months. The third dose is effective in mounting a significant immune response, but its durability is unknown.

**Objective:**

To compare antibody waning after second and third doses and estimate the association of antibody kinetics with susceptibility to infection with the Omicron variant of SARS-CoV-2.

**Design, Setting, and Participants:**

In a prospective longitudinal cohort study in a tertiary medical center in Israel, health care workers who received the BNT162b2 vaccine were followed up monthly for IgG and neutralizing antibody levels. Linear mixed models were used to compare antibody titer waning of second and third doses and to assess whether antibody dynamics were associated with Omicron transmission. Avidity, T cell activation, and microneutralization of sera against different variants of concern were assessed for a subgroup.

**Exposure:**

Vaccination with a booster dose of the BNT162b2 vaccine.

**Main Outcomes and Measures:**

The primary outcome was the rate of antibody titer change over time, and the secondary outcome was SARS-CoV-2 Omicron variant infection, as confirmed by reverse transcriptase–polymerase chain reaction.

**Results:**

Overall, 4868 health care workers (mean [SD] age, 46.9 [13.7] years; 3558 [73.1%] women) and 3972 health care workers (mean [SD] age, 48.5 [14.1] years; 996 [74.9%] women) were followed up for 5 months after their second and third vaccine doses, respectively. Waning of IgG levels was slower after the third compared with the second dose (1.32%/d [95% CI, 1,29%/d to 1.36%/d] vs 2.26% [95% CI, 2.13%/d 2.38%/d]), as was waning of neutralizing antibody levels (1.32%/d [95% CI, 1.21%/d to 1.43%/d] vs 3.34%/d [95% CI, 3.11%/d to 3.58%/d]). Among 2865 health care workers assessed for Omicron incidence during an additional 2 months of follow-up, lower IgG peak (ratio of means 0.86 [95% CI, 0.80-0.91]) was associated with Omicron infection, and among participants aged 65 years and older, faster waning of IgG and neutralizing antibodies (ratio of mean rates, 1.40; [95% CI, 1.13-1.68] and 3.58 [95% CI, 1.92-6.67], respectively) were associated with Omicron infection. No waning in IgG avidity was observed 112 days after the third dose. Live neutralization of Omicron was lower compared with previous strains, with a geometric mean titer at the peak of 111 (95% CI, 75-166), compared with 942 (95% CI, 585-1518) for WT, 410 (95% CI, 266-634) for Delta; it demonstrated similar waning to 26 (95% CI, 16-42) within 4 months. Among 77 participants tested for T cell activity, mean (SD) T cell activity decreased from 98 (5.4) T cells/10^6^ peripheral blood mononuclear cells to 59 (9.3) T cells/10^6^ peripheral blood mononuclear cells.

**Conclusions and Relevance:**

This study found that the third vaccine dose was associated with greater durability than the second dose; however, Omicron was associated with greater resistance to neutralization than wild type and Delta variants of concern. Humoral response dynamics were associated with susceptibility to Omicron infection.

## Key Points

**Question:**

What is the durability of the immune response after 3 vaccine doses, and are antibody kinetics associated with SARS-CoV-2 Omicron infection?

**Findings:**

In this cohort study of 3972 health care workers, reduction in antibody levels 5 months after the third BNT162b2 vaccine dose was slower than after the second, while Omicron's neutralizing response was lower compared with other variants of concern. Peak antibody levels after the third dose were associated with Omicron infection.

**Meaning:**

This study found that the humoral response after the third vaccine dose was sustained for 5 months and that antibody kinetics were associated with Omicron infection.

## Teaser

This cohort study investigates antibody waning after second and third doses of the BNT162b2 (BioNTech/Pfizer) COVID-19 vaccine.

## Introduction

The SARS-CoV-2 pandemic continues to take the lives of thousands of people daily and cause major economic, social, and public health impairments 2 years after emerging.^1^ Several vaccines have been shown to be efficacious and effective against infection and severe disease of SARS-CoV-2, and vaccine rollout is expanding.^2,3,4,5^ BNT162b2 (BioNTech/Pfizer) was approved as a 2-dose vaccine with a 21-day interval between doses.^6^ Several studies have demonstrated waning of immune responses and vaccine effectiveness of 2 doses within 6 months.^7,8,9^ Further findings revealed that older individuals, men, and those with more comorbidities mounted lower levels of humoral immune response.^10^ This led the Israel Ministry of Health and to recommend a booster dose on July 29, 2021, which other countries also did later.^11,12,13^ This additional vaccination was associated with rapidly increased immune response,^14,15^ high effectiveness,^4,16^ and a superior immune response compared with the second dose.^15^ Moreover, it was shown to be necessary for efficient neutralization of the Omicron variant of concern (VOC).^17,18^ While many countries are now recommending a third dose, the durability of third dose outcomes is unknown.

We investigated the association of the immune response at 5 months after the third dose with Omicron infections by assessing the durability of the immune response to the third vaccine dose and analyzing susceptibility to clinical infection 5 months after vaccination. Furthermore, we investigated the immune response against various VOCs and compared it with the immune response kinetics after the second dose.

## Methods

This cohort study’s protocol was approved by the Institutional Review Board of the Sheba Medical Center, and written informed consent was obtained from all study participants. The Strengthening the Reporting of Observational Studies in Epidemiology (STROBE) reporting guideline was followed in this study.

### Study Design and Population

The Sheba HCW COVID Cohort was established in March 2020, when 15 480 Sheba HCWs were invited to join a sero-surveillance study. With the rollout of the COVID-19 vaccination campaign, monthly follow-up was offered to all participants. The recruitment and follow-up of this cohort have been reported in detail previously^9,15,19,20,21^

Briefly, all HCWs older than age 18 years were invited to join the study if they were SARS-CoV-2 naïve (ie, did not have a previous positive reverse transcriptase–polymerase chain reaction [RT-PCR] result or detectable anti–SARS-CoV-2 receptor binding domain [RBD] IgG before the first vaccine dose). Any HCW who was infected by SARS-CoV-2 during the study was removed from the cohort to a parallel cohort of HCWs who were recovered from COVID-19. Participants were asked to undergo a serology test once every 4 weeks.

Data on age and sex were available for all 3972 study participants. Overall, 2723 participants (68.6%) responded to an online questionnaire regarding comorbidities, including immunosuppression and body mass index (calculated as weight in kilograms divided by height in meters squared) (eTable 9 in the Supplement).

### Primary and Secondary Study Outcomes

The primary outcome was the rate of decline of anti–SARS-CoV-2 RBD IgG (baseline characteristics are presented in eTable 1 in the Supplement) and pseudoneutralization titers (baseline characteristics are presented in eTable 2 in the Supplement) after the third dose of the vaccine compared with after the second dose of the vaccine. Secondary outcomes included immunogenicity outcomes: T cell activation (baseline characteristics are presented in eTable 3 in the Supplement), avidity (baseline characteristics are presented in eTable 4 in the Supplement), and microneutralization (baseline characteristics are presented in eTable 5 in the Supplement) of various VOCs. An additional secondary outcome was a comparison of waning dynamics between individuals infected and not infected with the Omicron variant.

**Figure 1.**
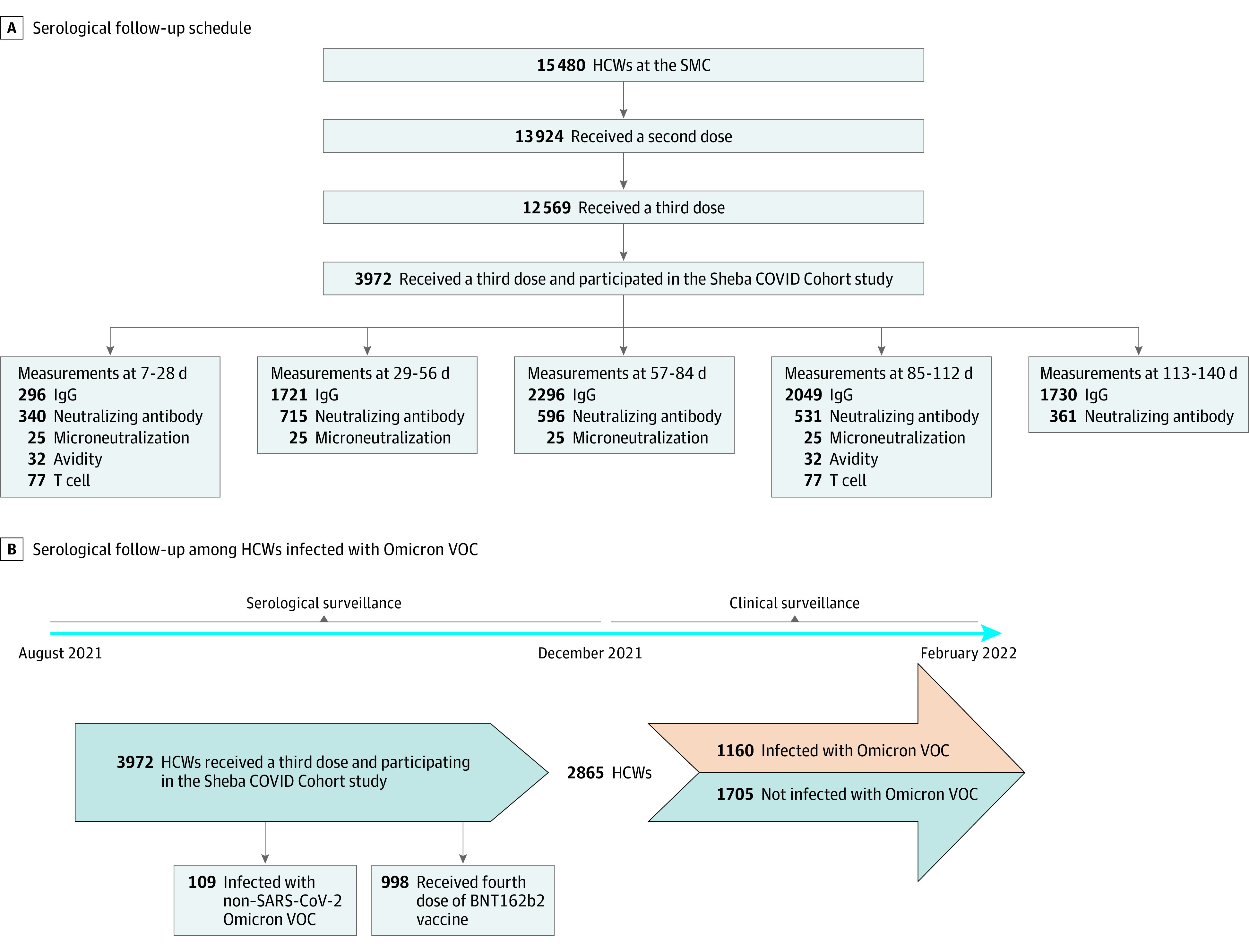
Recruitment of Participants, Testing, and Follow-up This study involved a prospective cohort of health care workers (HCWs) who received the third dose of the BNT162b2 vaccine and underwent at least 1 serologic assay afterward. A, The schedule for serological follow-up is presented. B, Serological follow-up of participants eventually infected with the Omicron variant is presented. Patients who received a fourth dose of vaccine and those who had an early breakthrough infection were censored. Serological follow-up of individuals who were infected that was obtained after infection was censored as well. IgG indicates IgG antibodies; SMC, Sheba Medical Center; VOC, variant of concern.

We compared dynamics of the immune response after the third dose (administered ≥5 months after the second dose) with those after the second dose. We included 3972 HCWs who received the third dose and had at least 1 serological test 7 to 140 days after the third dose of vaccine; the number of participants who had tests at different times during the study is presented in Figure 1A. Results after the third dose were compared with those among a cohort of 4868 of the same HCWs who had serology testing after the second dose previously described.^9^ Serology samples were collected between January 21, 2021, and December 29, 2021.

To further investigate if the rate of waning was associated with later breakthrough infections, we added 2 months of follow-up to assess the clinical outcome of participants during the Omicron surge, between December 15, 2021, and February 28, 2022. Only participants who were not infected by December 15, 2021, and did not receive a second booster (ie, fourth dose) during the study period were included in this secondary analysis. We compared dynamics of antibody waning after the third dose in individuals infected with COVID-19 during the Omicron surge vs those who remained uninfected during the follow-up period. The study design is depicted in Figure 1B. Variable definitions are described in eTable 10 in the Supplement.

### Serological Assays

#### IgG Antibody Assay

Samples from participants who were vaccinated were tested before receipt of the third dose using the SARS-CoV-2 RBD IgG assay (Beckman-Coulter or after receipt of the third dose using the SARS-CoV-2 IgG II Quant (Abbott) test performed according to the manufacturers’ instructions. All IgG Antibody levels were presented in binding antibody units (BAU) per World Health Organization standard measurements. Antibody detection testing is described in eMethods 3 in the Supplement.

#### Other Assays

To measure the quality of IgG antibodies, we used urea as a chaotropic reagent and tested the strength of interaction between the IgG and the viral antigen (the RBD) as was recently described.^15^ To test the overall neutralizing ability of each serum against the wild type (WT) virus and specifically to compare with neutralizing levels of Sheba HCWs after 2 and 3 vaccine doses, we used pseudovirus neutralization as previously described.^20^ Inclusion criteria for selecting the neutralizing antibody group are described in eMethods 2 in the Supplement. To compare the neutralizing capacity of Omicron and Delta variants after the third vaccine dose, a SARS-CoV-2 microneutralization assay with live virus was performed as previously described.^22^ Further details can be found in eMethods 4 in the Supplement. To investigate memory response, we isolated peripheral blood mononuclear cells (PBMCs) using Ficoll density gradient centrifugation. Using interferon γ enzyme–linked immunospot spot analysis, we measured SARS-CoV-2–specific T cell activation as described previously and detailed in eMethods 5 in the Supplement.^14^

### SARS-CoV-2 Detection

To identify infection during the study period, participants were asked to undergo a SARS-CoV-2 test, either RT-PCR (Seegene) or a rapid antigen test in case of exposure to an individual with a detected SARS-CoV-2 infection or the development of potential COVID-19 symptoms (eMethods 1 in the Supplement). During the Omicron surge (ie, December 15, 2021, to the end of the study, February 28, 2022), a routine weekly test was additionally requested. All RT-PCR SARS-CoV-2 tests conducted in the hospital or other settings were reported through a central reporting system.

### Statistical Analysis

The analysis of waning of IgG and neutralizing antibody levels was conducted with the same length of follow-up (140 days) for second and third vaccine doses. Abbott IgG levels after the second dose were imputed from Beckman-Coulter IgG levels using data on 215 selected serum samples not included in the HCW cohort using a cubic polynomial equation in log Beckman-Coulter level (*R* squared = 0.92) (eMethods 6 and 7 in the Supplement).

IgG tests taken before 30 days were used to estimate the peak IgG attained. Log antibody levels were modeled as constant up to 30 days after vaccination (ie, peak level), followed by a fixed effect linear decline from 30 days onward. Rates of decrease in antibody levels were analyzed using a linear mixed model in which log antibody level was the dependent variable and each individual’s peak level was modeled as a random effect. Separate models were run for second and third vaccine doses. For neutralizing antibody levels, the decrease starting at 70 days after the second vaccine dose but not the third dose was found to be slower than the initial rate of decline and was modeled accordingly; rates of decline reported are initial rates, and all rates are expressed on the original scale as percent decreases per day. Age (<45 years, 45-64 years, and ≥65 years) and sex were included as fixed-effect–adjusting covariates, and their interactions with peak level and rate of decline were included. For comparisons between doses, estimated mean peak levels, rates of decrease, and the level at day 140 after the third vaccine dose were standardized to the distribution of age and sex of individuals in the second dose cohort. Standard errors of estimates were model based for neutralizing antibodies, but for IgG, a bootstrap procedure was used to account for the extra uncertainty from imputing Abbott IgG levels for the second dose cohort (eMethods 6 and 7 in the Supplement). To compare kinetics of individuals who were infected vs those who were uninfected, an extra covariate indicating infection with Omicron (yes or no) was entered into the linear mixed model for individuals who received a third vaccine dose. Interactions between this covariate and peak level, rate of waning, and age group (<65 years or ≥65 years) were also included. From these models, ratios of mean peak levels and rates of waning in individuals who were infected vs those who were uninfected were computed separately for each age group. The linear mixed model analyses were implemented using the function lme found in the nlme package written in R statistical software version 3.6.0 (R Project for Statistical Computing).

Graphs were created using GraphPad Prism software version 9.0 (GraphPad Software). Correlations between IgG and neutralizing antibody levels for each period were assessed by Spearman rank correlation. Paired pre- and post-third vaccine dose avidity, neutralization, and T cell activation were compared using the Wilcoxon signed-rank test. Statistical analysis was performed using SAS statistical software version 9.4 (SAS Institute). We report all parameters with 95% CIs, with the interpretation as per the model fit.

## Results

### Study Population and Serologic Assays

In total, 8092 samples from 3972 HCWs who received 3 vaccine doses (mean [SD] age, 48.5 (14.1) years; 996 [74.9%] women) were collected from August 5, 2021, until December 29, 2021. Of these, clinical follow-up data were available for 2865 HCW, who were followed during the Omicron surge, between December 15, 2021, and February 28, 2022. (Figure 1). Samples from 4868 HCWs who received 2 doses of vaccine (mean [SD] age, 46.9 [13.7] years; 3558 [73.1%] women) were used for comparison. Demographic characteristics and data on coexisting conditions in study participants are provided in eTable 1 and eTable 2 in the Supplement.

### Waning of Humoral Response After Third vs Second Dose

**Figure 2.**
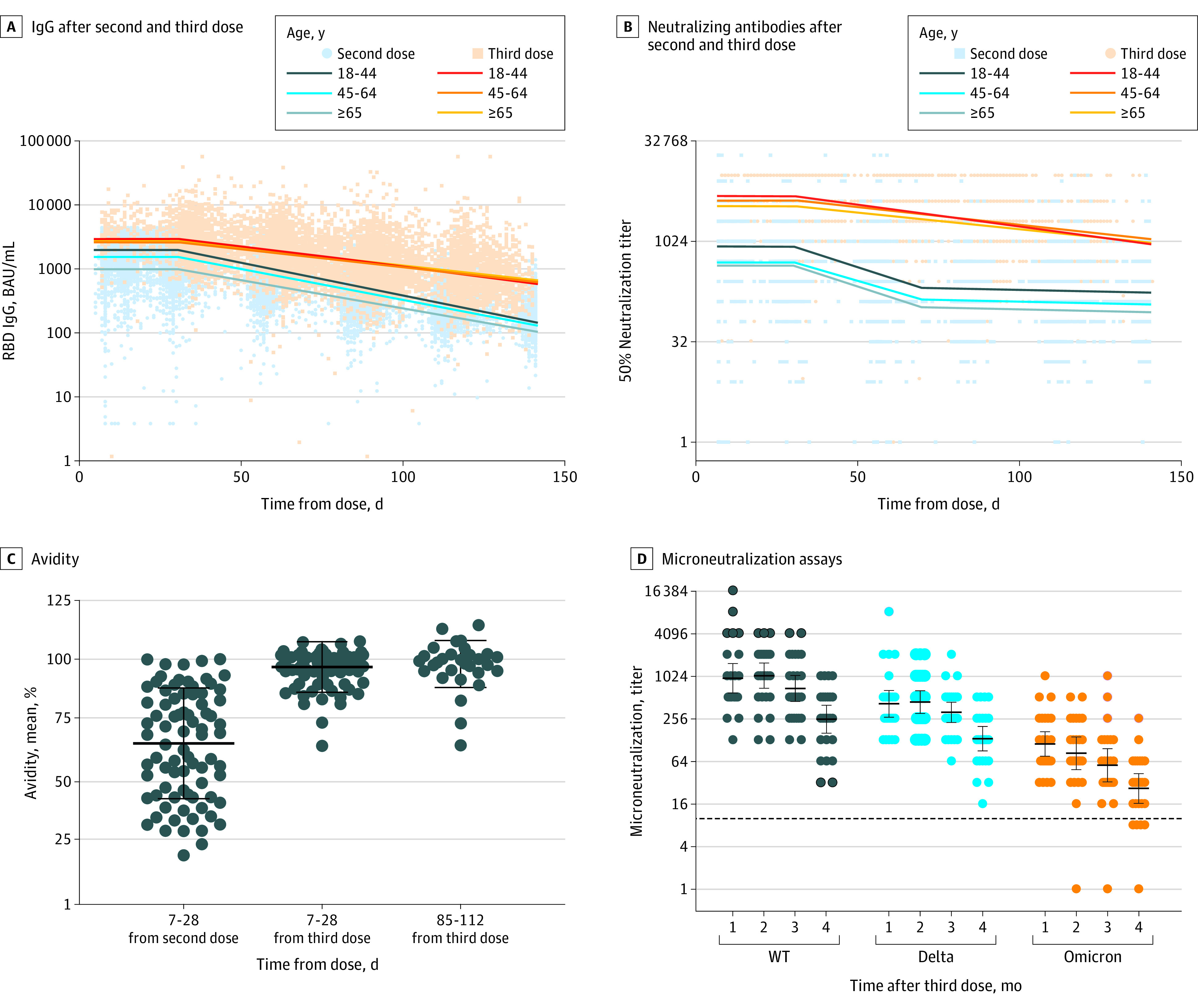
Humoral Response After Second and Third Doses A, The distribution of observed IgG antibodies after the second and third dose (points) and the expected geometric mean titer (GMT) as estimated by a model adjusted by age (lines) are presented. BAU indicates binding antibody unit; RBD, receptor binding domain. B, The distribution of observed neutralizing antibodies after the second and third dose (points) and the expected GMT as estimated by a model adjusted by age (lines) are presented. C, Antibody avidity 7 to 28 days after the second dose, 7 to 28 days after the third dose, and 85 to 112 days after the third dose are presented. Dots indicate observed results; horizontal black lines, means; whiskers, 95% CIs. D, The microneutralization assays against wild type (WT), Delta, and Omicron variants are presented, consisting of microneutralization of sera of 25 participants against WT, Delta, and Omicron variants at 1, 2, 3, and 4 months after the third dose of vaccine. Dashed horizontal line indicates cutoff for diagnostic positivity; dots, observed results; horizontal black lines, geometric means; whiskers, 95% CIs.

IgG waning was slower after the third dose of the vaccine (1.32%/d [95% CI, 1.29%/d to 1.36%/d]) vs after the second dose (2.26%/d [95% CI, 2.13%/d to 2.38%/d]) (Figure 2A and Table 1; eResults 1 in the Supplement). Neutralizing antibodies also had a slower rate of decrease after the third vaccine dose (1.32%/d [95% CI, 1.21%/d to 1.43%/d) vs 3.34%/d (95% CI, 3.11%/d to 3.58%/d). Neutralizing antibody kinetics after the third dose were constant and differed from kinetics after the second dose, where a substantial decrease continued beyond 70 days after the second vaccine dose (Figure 2B and Table 1). The mixed model analysis of variables associated with IgG and neutralizing antibody titers and rate of decrease is presented in eTable 7 and eResults 2 in the Supplement.

**Table 1.** IgG and Neutralizing Antibody Levels and Waning

	Levels, geometric mean (95% CI)		
	Second dose	Third dose	Ratio (third dose/second dose)
Peak^a^			
IgG, BAU	1675 (1542-1820)	2801 (2727-2878)	1.67 (1.53-1.82)
Neutralizing antibodies, titer	611 (557-671)	4315 (4051-4595)	7.06 (6.30-7.90)
Rate of waning, %/d			
IgG, BAU	2.26 (2.13-2.38)	1.32 (1.29-1.36)	0.59 (0.56-0.62)
Neutralizing antibodies, titer	3.34 (3.11-3.58)	1.32 (1.21-1.43)	0.39 (0.35-0.44)
Trough at 140 d			
IgG, BAU	140 (125-157)	650 (629-673)	4.64 (4.12-5.22)
Neutralizing antibodies, titer	132 (121-144)	1001 (895-1119)	7.57 (6.57-8.71)

Abbreviation: BAU, binding antibody unit.

^a^ IgG tests taken before 30 days were used to estimate the peak IgG attained.

A correlation and constant regression association in all study periods were observed between IgG and neutralizing antibodies (Spearman rank correlation between 0.59 and 0.74) after the third dose (eFigure 1 in the Supplement). Avidity was tested on a subgroup of 32 participants 1 and 4 months after the third vaccine dose and compared with outcomes 1 month after the second dose. Baseline characteristics of this population are described in eTable 4 in the Supplement. Mean (SD) avidity 1 month after the second dose was 65.7% (0.2%), increasing to 97.4% (0.1%) 1 month after the third vaccine dose and 98.04% (0.1%) 4 months after the third dose (Figure 2C; eTable 6 in the Supplement). No significant change in avidity was found at 6 months after the second dose (eFigure 2 in the Supplement). Observed results of the study population by time since the third vaccine dose are described in eTable6 in the Supplement.

### Association Between IgG Antibody Levels and Susceptibility to Omicron Infection

To assess if the rate of antibody decline was associated with Omicron infection, we followed up clinical outcomes of participants during the Omicron surge (December 15, 2021, to February 28, 2022). A random sample of 150 sequenced samples from this period consisted of 100% Omicron variant. A total of 2865 of 3972 HCWs who were not infected before the beginning of follow-up and did not receive the fourth dose were followed up during this period (Figure 1B). Overall 1160 of these HCWS (40.5%) were infected with SARS-COV-2 during this period. The time between the third vaccine dose and Omicron transmission varied among participants, ranging from 82 to 201 days, with a mean (SD) of 147.6 (20.6 days [95% CI, 106.4 days to 188.8 days]).

**Figure 3.**
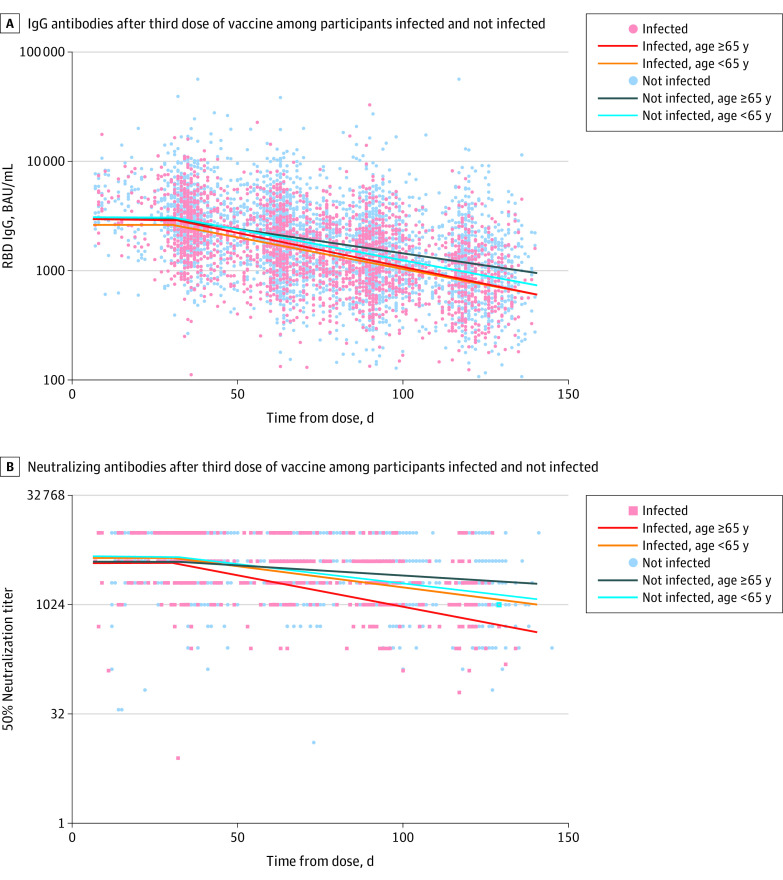
Distribution of Antibodies 150 Days After Third Doses Among Individuals Infected and Not Infected A, The distribution of observed IgG antibodies after the third dose among participants eventually infected and those who remained uninfected (points) and the expected geometric mean titer as estimated by a model adjusted by age are presented. B, The distribution of observed neutralizing antibodies after the third dose among participants eventually infected and those who remained uninfected (points) and the expected geometric mean titer as estimated by a model adjusted by age. BAU indicates binding antibody unit; RBD, receptor binding domain.

Demographics of individuals who were infected vs those not infected are described in eTable 8 in the Supplement; those infected were younger, with a mean age of 43.8 years (95% CI, 43.1 years to 44.5 years) vs 46.1 years (95% CI, 45.5 years to 46.7 years) among those who were not infected. In linear mixed model analysis, participants who were infected with COVID-19 at the time of the Omicron variant surge had a lower IgG peak after the third dose, at 2659 BAU (95% CI, 2528 BAU to 2797 BAU) vs 3107 BAU (95% CI, 2983 BAU to 3236 BAU) among those who were not infected (ratio of means between those infected and not infected, 0.86 [95% CI, 0.80-0.91]) (Table 2). Additionally, for participants aged 65 years or older, the rates of decrease for IgG and neutralizing antibodies were faster among HCWs who were infected. The rate of decrease of IgG antibodies was 1.39%/d (95% CI, 1.17%/d to 1.62%/d) among those who were infected vs 0.99%/d (95% CI, 0.89%/d to 1.10%/d) among those who were not infected, with a ratio of means of 1.40 (95% CI, 1.13-1.68). In addition, the rate of decrease of neutralizing antibodies was 1.86%/d (95% CI, 0.99%/d to 2.72%/d) among those who were infected vs 0.52%/d (95% CI, 0.14%/d to 0.90%/d) among those who were not infected, with a ratio of means of 3.58 (95% CI, 1.92-6.67) (Figure 3).

**Table 2.** Humoral Peak Levels, Rates of Decrease, and Ratios by Infection Status

Age group	Humoral levels, mean (95% CI)^a^	
	IgG antibodies, BAU	Neutralizing antibodies, titer
All ages		
Peak level		
Infected	2659 (2528-2797)	4564 (4047-5149)
Not infected	3107 (2983-3236)	4654 (4250-5097)
Ratio	0.86 (0.80-0.91)	0.98 (0.84-1.14)
Rate of decrease, %/d		
Infected	1.33 (1.28-1.38)	1.40 (1.17-1.62)
Not infected	1.26 (1.22-1.31)	1.13 (0.97-1.29)
Ratio	1.05 (1.00-1.11)	1.23 (0.97-1.50)
<65 y		
Peak level		
Infected	2633 (2503-2769)	4599 (4061-5209)
Not infected	3118 (2988-3255)	4784 (4334-5286)
Ratio	0.84 (0.79-0.90)	0.96 (0.82-1.13)
Rate of decrease, %/d		
Infected	1.32 (1.27-1.38)	1.33 (1.10-1.55)
Not infected	1.29 (1.24-1.33)	1.22 (1.05-1.40)
Ratio	1.03 (0.97-1.08)	1.09 (0.85-1.33)
≥65 y		
Peak level		
Infected	2964 (2300-3818)	4296 (2853-6469)
Not infected	2981 (2624-3386)	3853 (3077-4825)
Ratio	0.99 (0.75-1.32)	1.11 (0.70-1.78)
Rate of decrease, %/d		
Infected	1.39 (1.17-1.62)	1.86 (0.99-2.72)
Not infected	0.99 (0.89-1.10)	0.52 (0.14-0.90)
Ratio	1.40 (1.13-1.68)	3.58 (1.92-6.67)

Abbreviation: BAU, binding antibody unit.

^a^ Ratios were considered statistically significant when 95% CIs did not cross 1.

### Kinetics of SARS-COV-2 Cellular Immunogenicity After Third Vaccine Dose

T cell activity was tested for 77 participants 7 to 28 days after the third dose (at peak) and 85 to 112 days after the third dose (at trough). Baseline characteristics of this population are described in eTable 3 in the Supplement. Mean (SD) T cell activity at the peak was 98 (5.4) activated T cells/10^6^ PBMCs and decreased to 59 (9.3) activated T cells/10^6^ PBMCs within 3 to 5 months. During this time, the percentage of participants with no T cell response (no reaction on the Elispot) increased from 7 participants (9.1%) to 16 participants (20.8%). The dynamics of IgG and neutralizing antibodies tested in this group were similar to those of the full cohort (eTable 6 and eFigure 3 in the Supplement)

### Live Microneutralization of Omicron vs Other VOCs

Serum samples from 25 randomly selected individuals with 4 consecutive monthly samples were tested. Baseline characteristics of this population are described in eTable 5 in the Supplement. The neutralization geometric mean titer at the peak was 942 (95% CI, 585-1518) for WT, 410 (95% CI, 266-634) for Delta, and 111 (95% CI, 75-166) for Omicron. For all tested strains, similar waning within 4 months was observed (3-fold to 4-fold decrease) (Figure 2D; eTable 5 in the Supplement), reaching geometric mean titers of 249 (95% CI, 158-391), 131 (95% CI, 88-197), and 26 (95% CI, 16-42) for WT, Delta, and Omicron, respectively.

## Discussion

In this cohort study, we found a significantly slower waning of humoral response after the BNT162b2 third dose vaccine compared with after the second dose; however, a high rate of infection (40%) was seen during 2 months of the Omicron surge among HCWs vaccinated with a booster dose 5 to 6 months earlier. In addition, lower peak IgG levels were associated with susceptibility to Omicron infection, as was a faster decay of IgG levels, particularly among individuals aged 65 years or older. Finally, we observed that the waning of neutralization against the Omicron variant was similar to that against other strains and was consistently lower than against Delta and WT during 4 months of follow-up.

Two doses of the BNT162b2 vaccine elicit a rapid induction of humoral response^20^ followed by significant antibody waning,^7,9,23^ which was associated with approximately 30-fold and 6-fold lower IgG and neutralizing antibody levels, respectively, 6 months after the second dose.^9^ The superior humoral response after the third compared with the second dose was associated with in an increase in not only the quantity (IgG levels), but also the quality (avidity) of IgG antibodies.^15^ The durability of neutralizing titers after the third vaccine dose may, therefore, be associated with the persistence of high-quality IgG antibodies observed in our study for at least 4 months. With time and possible additional vaccine doses, future studies should continue to examine the interplay between quantity and quality of IgG antibodies given that these are crucial factors associated with vaccine efficacy.

Previous studies have shown that antibody levels are correlated with protection from infection,^24,25^ yet the threshold for protection is unknown because defining it would require measuring antibody levels just before infection. Alternatively, the rate of waning is more obtainable, and thus we examined whether this could be a factor associated with susceptibility to infection. Our results showed that at least in individuals aged 65 years or older, the rate of immune response waning was associated with Omicron transmission. Those aged 65 years or older who were eventually infected by Omicron had a steeper decrease in neutralizing and IgG antibody titers. Factors associated with variation in the rate of decrease among individuals of the same age group have yet to be studied. A future model estimating the risk of an individual being infected based on peak antibody levels and the rate of their decline could potentially estimate the individuals who should receive additional boosters and when they should receive them.

We and others have previously found that neutralizing antibody levels after 3 vaccine doses were approximately 4-fold to 10-fold lower against the Omicron compared with WT and Delta VOCs.^18,26^ Here, we reported a similar waning of direct microneutralization against different VOCs within 4 months after the third dose. This suggests that within a few months of waning, the neutralization efficacy of Omicron may be insufficient to prevent infections. Indeed, 2 studies^27,28^ found that protection against Omicron infections after a third mRNA vaccine dose waned over time. During the Omicron surge in Israel, which began 4 months after third dose vaccine rollout, a high rate of Omicron infections was reported,^29^ raising suspicion that immunogenicity toward this variant was decreased despite relatively high IgG and neutralizing antibody levels. Overall, these results suggest that the relative resistance of the Omicron variant to the humoral response induced even by a full vaccine series may be the major factor associated with a high transmission rate.

We have recently found^30^ that a second booster (fourth dose) was associated with IgG and neutralizing antibody levels similar to those induced by the first booster.^30^ However, we found that it was not associated with effective prevention of Omicron mild and asymptomatic infections among HCWs.^30^ However, a second booster was efficacious in protecting against severe disease and death compared with 1 booster dose.^4,31^ Despite the relatively slow waning of the immune response observed in this study, the lack of protection from infection by emerging SARS-CoV-2 variants suggests that repeated boosters of currently available vaccines may have reached a limit of protective outcomes in young and healthy populations. These results additionally suggest that the timing of second and third boosters in such populations should be further considered and studied. Our results in this study suggest that some populations may be more prone to Omicron infections and that, therefore, an updated Omicron vaccine could be advantageous. Indeed, preliminary data suggested that a bivalent Omicron BA.1 and prototype booster vaccine were associated with higher Omicron BA.1 and BA.4 and 5 neutralizing titers compared with the prototype vaccine alone.^32,33^ This result suggests that vaccine strain updates may be associated with protection against infection. Future studies should assess the effectiveness of a modified vaccine against infection of Omicron and other new emerging variants.

### Limitations

This study has several limitations. First, given that all participants were HCWs, they were primarily women and relatively younger and healthier than the general population, thus potentially limiting the study’s generalizability. Second, despite being validated on 215 samples before comparison, tests after the second dose were performed using a different assay and were thus imputed to be comparable with tests after the third dose. Although unlikely, this may potentially be a source of bias. Third, we have investigated the response only to the BNT162b2 vaccine, while the durability after other vaccines may be different. Fourth, the study period after waning was relatively short. Further studies to assess waning after longer periods are needed. Furthermore, given that participants were not blinded to their serology testing, those who had lower serological markers could have potentially had a lower threshold for RT-PCR testing, and this may have created bias. However, due to the outstanding surge in infections, all HCWs, were encouraged to obtain a weekly RT-PCR SARS-COV test during the study period in addition to testing after exposure or due to symptoms, regardless of their serological tests.

## Conclusions

Numerous studies have found that mRNA vaccines played a major role in protecting the world population against COVID-19. This cohort study found that the humoral response after a third dose was sustained for months with a minor decrease in antibody levels and that antibody levels were associated with infection with the Omicron variant, and thus, infection may potentially be estimated. Nevertheless, our results suggest that the humoral response generated by vaccination may not be enough to protect against Omicron infection. BNT162b2 booster doses have been found to be protective against severe disease and mortality, yet if reducing transmissibility and achieving herd immunity is the goal, our results suggest that a different vaccination strategy may be required.

## Notes

Supplement. **eMethods 1.** Polymerase Chain Reaction Testing **eMethods 2.** Inclusion Criteria for Selecting Neutralizing Antibody Group **eMethods 3.** Antibody Detection Testing **eMethods 4.** SARS-CoV-2 Microneutralization **eMethods 5.** Memory Immune Response **eMethods 6.** Imputation of Binding Antibody Units and IgG Linear Mixed Model **eMethods 7.** Neutralizing Antibody Linear Mixed Model **eResults 1.** IgG Linear Mixed Models **eResults 2.** Neutralizing Antibody Linear Mixed Model **eTable 1.** Baseline Characteristics of IgG Study Population **eTable 2.** Baseline Characteristics of Neutralizing Antibodies Study Population **eTable 3.** Baseline Characteristics of T Cell Activation Population **eTable 4.** Baseline Characteristics of Avidity Study Population **eTable 5.** Baseline Characteristics of Microneutralization Study Population **eTable 6.** Observed Results of Study Population by Time Since Third Dose of Vaccination **eTable 7.** Mixed Model Analysis of Variables Associated with IgG and Neutralizing Antibody Titers After Second Vaccine Dose **eTable 8****.** Baseline Characteristics of Participants Eventually Infected vs Those Not Infected **eTable 9.** Computer-Based Questionnaire **eTable 10.** Variable Definitions **eFigure 1.** Correlation Between Neutralizing Antibody Titers and IgG Antibody Titers by Month **eFigure 2.** Antibody Avidity after Second Dose **eFigure 3.** Immunogenicity of Subgroup of 77 Participants
eReferences.
