# Supplementary material for: Durability of Immune Response After COVID-19 Booster Vaccination and Association With COVID-19 Omicron Infection
Source: JAMA Netw Open. 2022 Sep 15;5(9):e2231778. doi: 10.1001/jamanetworkopen.2022.31778 (PMC9478782; doi:10.1001/jamanetworkopen.2022.31778)
Supplement: Supplement. — eMethods 1. Polymerase Chain Reaction Testing eMethods 2. Inclusion Criteria for Selecting Neutralizing Antibody Group eMethods 3. Antibody Detection Testing eMethods 4. SARS-CoV-2 Microneutralization eMethods 5. Memory Immune Response eMethods 6. Imputation of Binding Antibody Units and IgG Linear Mixed Model eMethods 7. Neutralizing Antibody Linear Mixed Model eResults 1. IgG Linear Mixed Models eResults 2. Neutralizing Antibody Linear Mixed Model eTable 1. Baseline Characteristics of IgG Study Population eTable 2. Baseline Characteristics of Neutralizing Antibodies Study Population eTable 3. Baseline Characteristics of T Cell Activation Population eTable 4. Baseline Characteristics of Avidity Study Population eTable 5. Baseline Characteristics of Microneutralization Study Population eTable 6. Observed Results of Study Population by Time Since Third Dose of Vaccination eTable 7. Mixed Model Analysis of Variables Associated with IgG and Neutralizing Antibody Titers After Second Vaccine Dose eTable 8. Baseline Characteristics of Participants Eventually Infected vs Those Not Infected eTable 9. Computer-Based Questionnaire eTable 10. Variable Definitions eFigure 1. Correlation Between Neutralizing Antibody Titers and IgG Antibody Titers by Month eFigure 2. Antibody Avidity after Second Dose eFigure 3. Immunogenicity of Subgroup of 77 Participants eReferences. [file jamanetwopen-e2231778-s001.pdf]

Supplemental Online Content

Gilboa M, Regev-Yochay G, Mandelboim M, et al. Durability of immune response after COVID-19 booster vaccination and association with COVID-19 Omicron infection. *JAMA Netw Open*. 2022;5(9):e2231778. doi:10.1001/jamanetworkopen.2022.31778

eMethods 1. Polymerase Chain Reaction Testing

eMethods 2. Inclusion Criteria for Selecting Neutralizing Antibody Group

eMethods 3. Antibody Detection Testing

eMethods 4. SARS-CoV-2 Microneutralization

eMethods 5. Memory Immune Response

eMethods 6. Imputation of Binding Antibody Units and IgG Linear Mixed Model

eMethods 7. Neutralizing Antibody Linear Mixed Model

eResults 1. IgG Linear Mixed Models

eResults 2. Neutralizing Antibody Linear Mixed Model

eTable 1. Baseline Characteristics of IgG Study Population

eTable 2. Baseline Characteristics of Neutralizing Antibodies Study Population

eTable 3. Baseline Characteristics of T Cell Activation Population

eTable 4. Baseline Characteristics of Avidity Study Population

eTable 5. Baseline Characteristics of Microneutralization Study Population

eTable 6. Observed Results of Study Population by Time Since Third Dose of Vaccination

eTable 7. Mixed Model Analysis of Variables Associated with IgG and Neutralizing Antibody Titers After Second Vaccine Dose

eTable 8. Baseline Characteristics of Participants Eventually Infected vs Those Not Infected

eTable 9. Computer-Based Questionnaire

eTable 10. Variable Definitions

eFigure 1. Correlation Between Neutralizing Antibody Titers and IgG Antibody Titers by Month

eFigure 2. Antibody Avidity after Second Dose

eFigure 3. Immunogenicity of Subgroup of 77 Participants

eReferences.

This supplemental material has been provided by the authors to give readers additional information about their work.

© 2022 Gilboa M et al. *JAMA Network Open*.

eMethods 1. Polymerase Chain Reaction Testing

Hospital personnel were tested in several scenarios: upon every symptom suspected to be COVID-19, following exposure to a positive COVID-19 contact (hospital or community contacts).

For quantitative RealTime-PCR (qRT-PCR), nasopharyngeal swabs were placed in 3mL of universal transport medium (UTM) or viral transport medium (VTM). The test was performed according to manufacturers' instructions on various platforms: Allplex™ 2019-nCoV (Seegene, S. Korea), NeuMoDx™ SARS-CoV-2 assay (NeuMoDx™ Molecular, Ann Arbor, Michigan), Xpert®, Xpress SARS-CoV-2 (Cepheid, Sunnyvale, CA, USA).

© 2022 Gilboa M et al. *JAMA Network Open*.

eMethods 2. Inclusion Criteria for Selecting Neutralizing Antibody Group

1. Age ≥65
2. Body mass index ≥30
3. Pregnancy
4. Allergy
5. Hypertension
6. Diabetes
7. Dyslipidemia
8. Heart disease
9. Lung disease
10. Kidney disease
11. Liver disease
12. Autoimmune disease
13. Immunosuppression

Additionally, 50% of healthy health care workers were randomly selected for the neutralizing antibody subgroup.

© 2022 Gilboa M et al. *JAMA Network Open*.

eMethods 3. Antibody Detection Testing

SARS-CoV-2 IgG Assay

Samples from vaccinated participants were tested before receipt of the third dose using the SARS-CoV-2 Receptor Binding Domain (RBD) IgG assay (Beckman-Coulter, CA, U.S.A.), or after receipt of the third dose using the SARS-CoV-2 IgG II Quant (Abbott, IL, USA) test. These commercial tests were performed according to the manufacturer's instructions. To present all IgG Antibody levels in Binding Antibody Units (BAU) per the World Health Organization (WHO) standard measurements we imputed the Abbott-based BAU values from the Beckman-Coulter assay results, based on an independent sample of individuals with both Abbott BAU and Beckman-Coulter levels.

Avidity

To measure the quality of IgG antibodies we used urea as a chaotropic reagent and test the strength of interaction between the IgG and the viral antigen (the RBD). Specifically, a 96-well microtiter Polysorb plate (Nunc, Thermo, Denmark) was coated overnight at 4°C with 50µl per well of 1µg/ml of RBD antigen. After blocking with 5% skimmed milk at 25°C for 60 minutes, serum samples were diluted at 1:100, 1:400, and 1:1000 with 3% skimmed milk and added to antigen-coated wells. The plate was incubated at 25°C for 120 minutes, and following washing each sample, was incubated either with the addition of 6M urea or PBS for 10 min. After washing, a goat anti-human IgG horseradish peroxidase (HRP) conjugate (Jackson ImmunoResearch, PA, USA Code: 109-035-088) (diluted 1:15000) was be added to each well for 60 min. After washing, incubation of TMB Substrate Solution (Abcam) for 5 min, and the addition of stop solution (2N HCl), the OD of each well was measured at 450nm using a microplate reader (Sunrise, Tecan). Avidity index was calculated as the ratio (in percentage) between sample OD with 6M urea and sample OD with PBS.

SARS-CoV-2 Pseudovirus (psSARS-2) Neutralization Assay

To test the overall neutralizing ability of each serum against the WT virus and specifically to compare with neutralizing levels of the SHEBA HCW following one, two, and three vaccine doses we used Pseudovirus (psSARS-2) Neutralization as previously described[1]. SARS-CoV-

© 2022 Gilboa M et al. *JAMA Network Open*.

2 Pseudo-virus (psSARS-2) Neutralization Assay was performed using a propagation-competent VSV-spike which was shown to be highly correlative to authentic SARS-CoV-2 virus micro-neutralization assay. Following titration, 100 focus forming units (ffu) of psSARS-2 were incubated with 2-fold serial dilution of heat-inactivated (56°C for 30 min) tested sera. After incubation for 60 min at 37°C, the virus/serum mixture was transferred to Vero E6 cells that have been grown to confluency in 96-well plates and incubated for 90 min at 37°C. After the addition of 1% methylcellulose in Dulbecco's modified eagle's medium (DMEM) with 2% of fetal bovine serum (FBS), plates were incubated for 24hr and 50% plaque reduction titer was calculated by counting green fluorescent foci using a fluorescence microscope. Sera not capable of reducing viral replication by 50% at 1 to 16 dilution or below was considered non-neutralizing.

© 2022 Gilboa M et al. *JAMA Network Open*.

eMethods 4. SARS-CoV-2 Microneutralization

To compare the neutralizing capacity of omicron and delta variants following the third vaccine dose a SARS-CoV-2 micro-neutralization assay with live virus was performed as previously described [2,3].VERO-E6 cells at a concentration of $20*10^5$/well were seeded in sterile 96-wells plates with 10% FCS MEM-EAGLE medium and stored at 37ºC for 24 hours. One hundred TCID50 of Wild Type, Beta, Delta, and Omicron SARS-CoV-2 isolates were incubated with inactivated sera diluted 1:10 to 1:16,384 in 96 well plates for 60 minutes at 37ºC. Virus-serum mixtures were added to the Vero E-6 cells and incubated for five days at 37ºC after which Gentian violet staining (1%) was used to stain and fix the cell culture layer. Neutralizing dilution of each serum sample was determined by identifying the well with the highest serum dilution without observable cytopathic effect. A dilution equal to 1:10 or above was considered neutralizing.

© 2022 Gilboa M et al. *JAMA Network Open*.

eMethods 5. Memory Immune Response

To investigate the memory response we isolated peripheral blood mononuclear cell (PBMC) using Ficoll density gradient centrifugation and analyzed T cell activation as described previously [4].

T cell activation was assessed by IFN-γ ELISpot assay. Specifically, IFN- γ -secreting cells were enumerated using Elispot IFN-γ kits (IFN-γ kit, AID Autoimmun Diagnostika GmbH, Strassberg, Germany) according to manufacturer instructions. For antigen stimulation, 50 µl of SARS-CoV-2 peptide pools (S-complete, Miltenyi Biotech) was used. Test medium was used as negative control and Phytohaemagglutinin (PHA) was used as a positive control. IFN-γ-secreting cells frequency was quantified using the AID ELISpot Reader (Strassberg, Germany). The unspecific background (mean SFU from negative control wells) was subtracted from experimental readings.

© 2022 Gilboa M et al. *JAMA Network Open*.

eMethods 6. Imputation of Binding Antibody Units and IgG Linear Mixed Model

S6.1 Imputation of Binding Antibody Units based on Beckman-Coulter Assay Results

We developed a method for imputing Abbott IgG levels from Beckman-Coulter IgG levels using data on 215 selected serum samples, taken from individuals who had not received a booster dose and were not included in the HCW cohort and were measured by both methods. We fitted a cubic polynomial regression model where the log (to the base e) of IgG measured in BAU units by the Abbott kit was regressed on the log (to the base e) of IgG measured by the Beckman-Coulter kit, its squared value, and its cubed value. The fitted regression equation, using the glm procedure in R, was:

logIGG_Abbott =
$4.506 + 0.6634 \times \text{logIGG\_Beckman} - 0.0852\times (\text{logIGG\_Beckman})^2 + 0.0403\times (\text{logIGG\_Beckman})^3$

Output from the glm procedure in R

```
Coefficients:
                Estimate Std. Error t value Pr(>|t|)    
(Intercept)     4.506257   0.039815 113.180  < 2e-16 ***
logBeck         0.663432   0.042540  15.595  < 2e-16 ***
I(logBeck^2)   -0.085175   0.020513  -4.152 4.78e-05 ***
I(logBeck^3)    0.040341   0.007083   5.695 4.10e-08 ***
---
Signif. codes:  0 '***' 0.001 '**' 0.01 '*' 0.05 '.' 0.1 ' ' 1

Residual standard error: 0.4377 on 211 degrees of freedom
Multiple R-squared:  0.9187,   Adjusted R-squared:  0.9176
```

A simple linear model has an R-squared of 0.905, compared to 0.919 for the cubic polynomial. One can see from the figure below that the cubic polynomial fits the data much better at the lower and upper ends of the scale.

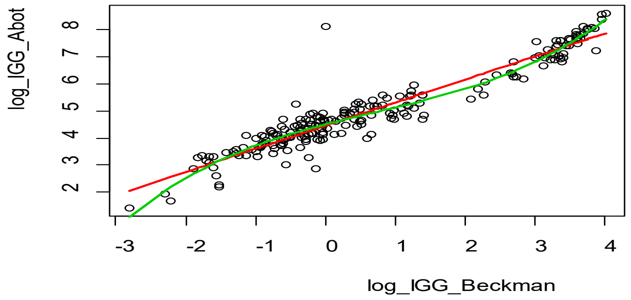

© 2022 Gilboa M et al. *JAMA Network Open*.

The regression equation shown above was used to impute the values of Abbott BAU for samples taken after the second vaccine dose. To avoid extrapolation, any Beckman-Coulter IgG value that was lower than the minimum value of the calibration sample (0.06) was assigned to that minimum (occurring in 0.1% of the HCW sample) and any value above the maximum value (55.29) was assigned to that maximum (occurring in 2.2% of the HCW sample). Abbott BAU levels following the third vaccine dose were measured directly and did not require imputation.

S6.2 Linear Mixed Model for IgG Level Kinetics Following Second and Third Vaccine Doses

We modeled the natural log-transformed IgG in a mixed-effects linear model with subject-level random effects. Separate models were run for the second and third vaccine doses. We fit a linear slope from 30 days after vaccination onwards. At the same time, using the statistical m described below, IgG tests taken before 30 days were used to estimate the "peak" IgG attained. We fitted a model that included age group (<45, 45-64, ≥65), sex, time (measured in days from day 30 post-vaccine onwards; all measurements before day 30 were counted as day 0), and all age-sex-time interactions (including the 3-way interaction) as fixed effects, and subject-level random intercept. For the third dose, the three-way interaction between age, sex, and time was small and not statistically significant, and therefore omitted.

In summary, for the second dose, we used the following statistical model:

$$\log IgG_{ij} = M + s_i + (\beta_d + b_{di})\,(days_{ij} - 30)_+ + \beta_G\, G_i + \beta_A\, A_i + \beta_{GA}\, G_i A_i + \beta_{Gd}\, G_i (days_{ij} - 30)_+ + \beta_{Ad}\, A_i (days_{ij} - 30)_+ + \beta_{GAd}\, G_i A_i (days_{ij} - 30)_+ + \varepsilon_{ij}\,,$$

where IgG_{ij} is the IgG level of subject *i* measured on the jth occasion, M is an overall intercept, $(days_{ij} - 30)_+$ is the time of measurement since 30 days post-vaccination and is set equal to 0 for the first thirty days post-vaccination, G_i is the gender of subject *i* (coded 0 for female and 1 for male), A_i is vector denoting the age-group of subject *i* (coded [0,0] for

<45y, [1,0] for 45-64y and [1,1] for 65+y), the β's are the regression coefficients, and s_i , b_{di}, ε_{ij} are the subject random intercept, the subject random slope, and the residual error, respectively, all assumed to be independently distributed normally with mean zero, the residual error independently of the intercept and slope. The same model was used for levels following the third dose, except that the term $\beta_{GAd}\ G_i A_i (days_{ij} - 30)_+$ was omitted.

Expected peak IgG levels, rates of decline, and level at day 140 following vaccine for each age-sex profile were estimated from the coefficients obtained from the model fit and averaged over the cohorts, with weights according to the their sample proportions. Specifically, for the second dose, the log peak for subject i 's age-sex profile was estimated from $M + \beta_G\ G_i + \beta_A\ A_i + \beta_{GA}\ G_i A_i$, the rate of change per day on the log scale from $\beta_d + \beta_{Gd}\ G_i + \beta_{Ad}\ A_i + \beta_{GAd}\ G_i A_i$, and the log level at 140 days from $M + 110\ \beta_d + \beta_G\ G_i + \beta_A\ A_i + 110\ \beta_{Gd}\ G_i + 110\beta_{Ad}\ A_i + 110\ \beta_{GAd}\ G_i A_i$. The same expressions were used for kinetics parameters following the third dose, except that the terms involving β_{GAd} were omitted.

For comparisons between the kinetics following the second and third doses, the weighted averages of peak level, rate of decline and level at 140 days for the third vaccine dose were standardized to the distribution of age and sex of those in the second dose cohort. Ratios of these averaged parameters for the third dose to the averaged parameters for the second dose were then computed together with a 95% confidence interval.

The confidence intervals were computed by first estimating the standard error of the ratio using the delta method $\left[SE\left(\frac{x}{y}\right) = \sqrt{\left(\frac{E(x)}{E(y)}\right)^2 \left(\frac{var(x)}{E(x)^2} + \frac{var(y)}{E(y)^2}\right)}\right]$, and then assuming that the sampling distribution of the estimate on the log scale was asymptotically normal, giving a confidence interval equal to estimated ratio ± 1.96 × standard error. Results for peak and level at 140 days were then translated back to the original scale.

© 2022 Gilboa M et al. *JAMA Network Open*.

This delta method assumes statistical independence of the numerator and denominator; for its computation, the method required estimates of the standard errors of the numerator and denominator of the ratio. These were computed as follows. For the denominator (the second dose estimate), a bootstrap procedure was used to account for the uncertainty due to the imputation: first a bootstrap of the calibration sample was taken and the imputation equation was re-computed; then a bootstrap of the HCW sample for the second dose was taken, the linear mixed model was run, and the age-sex standardized peak, slope or 14-day level was estimated; from the distribution of these parameters across the bootstrap samples, a standard error was computed. The standard error of the numerator (the third dose estimate) was derived directly from the model-based variance-covariance matrix of the regression coefficients estimates from the linear mixed model.

There are two assumptions in the above method that may not hold true. Firstly, the normality of the sampling distribution of the ratio, and secondly the statistical independence of the numerator and denominator. To check on whether these lead to inaccurate confidence intervals we also ran a method that was entirely based on bootstrap sampling and not dependent on the above assumptions. As previously, a bootstrap sample of the calibration sample was first taken and the calibration equation was recomputed and applied to the Beckman-Coulter readings following the second dose. This step was followed by taking a stratified bootstrap sampling of the HCW cohort using three strata: (i) those HCWs contributing to follow-up following only the second dose; (ii) those HCWs contributing to follow-up following only the third dose; and (iii) those HCWs contributing to follow-up following both the second and the third dose. Separate linear mixed models were conducted following each dose; for the second dose including strata (i) and (iii), and for the third dose including strata (ii) and (iii). The peak level, rate of decline and level at 140 days were computed for each dose as described above, and the ratio of third to second dose calculated

© 2022 Gilboa M et al. *JAMA Network Open*.

for each of these parameters. This bootstrap sampling was repeated 1000 times, and the confidence intervals for the ratios was computed using the bootstrap percentile method, after checking that the bootstrap distributions appeared symmetric. In the table below we compare the confidence intervals based on the delta method (that were reported in the paper) with those based on the bootstrap method. It can be seen that there is excellent agreement between the two methods, so we have retained the results from the delta method in our revised version of the paper.

Antibody Test	Parameter	Ratio 3rd to 2nd dose	95% CI Delta method	95% CI Bootstrap
IgG	Peak	1.67	1.53-1.82	1.54-1.83
	Rate of decline	0.59	0.56-0.62	0.55-0.63
	Level at 140d	4.64	4.12-5.22	4.10-5.26
Neutralizing	Peak	7.06	6.30-7.90	6.40-7.78
	Rate of decline	0.39	0.35-0.44	0.35-0.43
	Level at 140d	7.57	6.57-8.71	6.63-8.66

S6.3 Comparison between Omicron-Infected and Non-Infected persons concerning IgG Level Kinetics Following Third Vaccine Dose

To compare the kinetics of omicron-infected versus uninfected persons, an extra covariate indicating infection with omicron (yes/no) was entered into the linear mixed model for those who received a third vaccine dose. This approach seemed reasonable under the following circumstances. Infection status was determined during the period of the omicron surge in Israel, from December 15th 2021 to February 27th 2022, and those who had been infected before December 15th were excluded. The serological measurements were those made up to the end of December 31st 2021. Thus, the great majority of infections occurred after the last serological measurement used in this study, especially since the major omicron surge occurred in January 2022. There were 24 HCWs (0.8% of the total number included in this analysis and 2.0% of the number of infected HCWs included in this analysis) who developed an infection

© 2022 Gilboa M et al. *JAMA Network Open*.

before December 31st 2021. Serological measurements made after infection were not included in the analysis. Viewing infection status as a fixed covariate at the individual level, seems reasonable in view of the predominant separation of the period of antibody measurements from the period of infections.

Interactions between age (<65, ≥65), sex, time and omicron infection were tested and retained if significant at the 5% level. When a higher-order interaction was retained all its lower-order interactions were also retained. In this way, we included in the model all main effects and the following interactions: age-omicron-time; age-time; omicron-time; sex-time. From the parameter estimates of this model, ratios of average peak levels and rates of waning between omicron-infected and uninfected persons were computed separately for each age group. Standard errors of the ratios between omicron-infected and uninfected were computed for these parameters (peak level and rate of waning) using the delta method.

© 2022 Gilboa M et al. *JAMA Network Open*.

eMethods 7. Neutralizing Antibody Linear Mixed Model

We modeled log-transformed (to the base 2) neutralizing antibody (NeutAb) using a linear mixed-effects model similar to the model used for the IgG analysis (Supplementary Methods S6.2). Separate models were run for the second and third vaccine doses. The model included subject-level random effects. For the second dose, NeutAb kinetics over time was different from IgG kinetics. The decline started 30 days after vaccination as in the IgG data (see Supplementary Methods S6.2). However, the decline lasted only up to ~70 days after vaccination and then stabilized to a much slower decline. The change point at 70 days was chosen based on inspection of the fit of previous data without the change point, and examining AIC for models with a change point at 60, 70, 80, and 90 days. Therefore, we included in the model a separate slope from day 70 to day 140 post-vaccine. For the third dose, the slope after day 70 was not more gradual than up to day 70 and the decline was therefore modeled as a linear slope from day 30 onwards (as for IgG levels). Other main effects and interactions, including the interaction between fixed-effect factors with time in the period from day 30 to day 70, were the same as in the model for IgG levels, except that for the third dose, the interaction between age and sex was small and non-significant, and therefore omitted.

Average peak NeutAb levels, rates of decline, and level at day 140 following vaccine were estimated and compared between doses using the same methods as described in Supplementary Methods S6.2. Standard errors of estimates were model-based, and those ratios were based on the delta method.

To compare the kinetics of omicron-infected versus uninfected persons, the same methods were used for IgG levels (see Supplementary Methods S6.3), and the same interaction terms as for the IgG model were retained. From the parameter estimates of this model, ratios of average peak levels and rates of waning between omicron-infected and uninfected persons were computed separately for each age group. Standard errors of these ratios were computed

© 2022 Gilboa M et al. *JAMA Network Open*.

using bootstrap methods, because, with the smaller numbers in this analysis, the asymptotic delta method was thought less reliable.

© 2022 Gilboa M et al. *JAMA Network Open*.

eResults 1. IgG Linear Mixed Models

The results of fitting the linear mixed model for the IgG level kinetics following the second and third doses are shown below.

1._Model fixed parameter estimates for natural log IgG levels following **the second** dose (se denotes the standard error obtained by the bootstrap sampling; pval denotes the p-value obtained from the z-test based on est/se)

```
Fixed effects: ligg ~ agec * male * days30

                           est     se   pval
(Intercept)             7.6537 0.0491 0.0000
agec45-64              -0.2655 0.0346 0.0000
agec65+                -0.6006 0.1018 0.0000
male                   -0.1099 0.0470 0.0193
days30*                -0.0239 0.0008 0.0000
agec45-64:male         -0.0802 0.0695 0.2484
agec65+:male           -0.2567 0.1654 0.1207
agec45-64:days30        0.0017 0.0003 0.0000
agec65+:days30          0.0041 0.0009 0.0000
male:days30             0.0016 0.0004 0.0002
agec45-64:male:days30  -0.0011 0.0006 0.0533
agec65+:male:days30    -0.0026 0.0013 0.0448

* days 30 is defined as taking value 0 until day 30 and increasing from 0 to 110
linearly until 140.
```

2._Model fixed parameter estimates for natural log IgG levels following the **third** dose (Standard errors obtained from the model; p-values based on the t-test)

```
Fixed effects: ligg ~ agec * male + days30 * agec + male * days30

                      Value  Std.Error   DF  t-value p-value
(Intercept)        7.964272 0.02449155 4105 325.1845  0.0000
agec45-64         -0.029576 0.03284721 3919  -0.9004  0.3680
agec65+           -0.002495 0.05152954 3919  -0.0484  0.9614
male              -0.005061 0.04868796 3919  -0.1040  0.9172
days30            -0.014259 0.00025796 4105 -55.2775  0.0000
agec45-64:male    -0.044696 0.06771464 3919  -0.6601  0.5093
agec65+:male      -0.180259 0.08295398 3919  -2.1730  0.0298
agec45-64:days30   0.000880 0.00030912 4105   2.8453  0.0045
agec65+:days30     0.001064 0.00039535 4105   2.6922  0.0071
male:days30        0.001816 0.00032140 4105   5.6515  0.0000
```

3. Model fixed parameter estimates for comparing kinetics between **omicron-infected and uninfected persons** following the **third** dose. (Standard errors obtained from the model; p-values based on the t-test)

```
                     Value Std.Error   DF  t-value p-value
(Intercept)         8.0524    0.0232 2860 346.6659  0.0000
age65              -0.0394    0.0689 2860  -0.5717  0.5676
male               -0.0372    0.0405 2860  -0.9187  0.3583
omic               -0.1692    0.0338 2860  -5.0105  0.0000
days30             -0.0134    0.0002 2645 -56.4218  0.0000
age65:omic          0.1634    0.1486 2860   1.0998  0.2715
male:days30         0.0021    0.0004 2645   4.7262  0.0000
omic:days30        -0.0004    0.0004 2645  -0.9844  0.3250
age65:days30        0.0027    0.0006 2645   4.3296  0.0000
age65:omic:days30 -0.0037    0.0014 2645  -2.7325  0.0063
```

© 2022 Gilboa M et al. *JAMA Network Open*.

eResults 2. Neutralizing Antibody Linear Mixed Model

The results of fitting the linear mixed model for the neutralizing antibody (NeutAb) level kinetics following the second and third doses are shown below.

1. Model fixed parameter estimates for log (base 2) NeutAb levels following the **second** dose (Standard errors obtained from the model; p-values based on the t-test)

Fixed effects: lneut ~ agec * male * days30a + days70

	Value	Std.Error	DF	t-value	p-value
(Intercept)	9.9773	0.1143	2472	87.2604	0.0000
agec45-64	-0.9978	0.1600	1222	-6.2364	0.0000
agec65+	-0.9442	0.1764	1222	-5.3533	0.0000
male	-0.9563	0.2896	1222	-3.3021	0.0010
days30a*	-0.0559	0.0027	2472	-20.9726	0.0000
days70	-0.0035	0.0010	2472	-3.4490	0.0006
agec45-64:male	0.5844	0.4057	1222	1.4405	0.1500
agec65+:male	0.2030	0.3621	1222	0.5606	0.5752
agec45-64:days30	0.0120	0.0035	2472	3.4765	0.0005
agec65+:days30	0.0056	0.0040	2472	1.3977	0.1623
male:days30	0.0165	0.0062	2472	2.6670	0.0077
agec45-64:male:days30	-0.0246	0.0089	2472	-2.7716	0.0056
agec65+:male:days30	-0.0201	0.0080	2472	-2.5076	0.0122

* days30a is defined as taking value 0 until day 30, increasing from 0 to 40 linearly until day 70, and then remaining constant at 40 from day 70 to 140.

2. Model fixed parameter estimates for log (base 2) IgG levels following the **third** dose (Standard errors obtained from the model; p-values based on the t-test)

Fixed effects: ligg ~ days30 * agec + male * days30

	Value	Std.Error	DF	t-value	p-value
(Intercept)	12.3447	0.0832	1602	148.2980	0.0000
agec45-64	-0.2311	0.1016	974	-2.2754	0.0231
agec65+	-0.4501	0.1156	974	-3.8941	0.0001
days30*	-0.0231	0.0015	1602	-15.4331	0.0000
male	-0.4456	0.0951	974	-4.6857	0.0000
agec45-64:days30	0.0045	0.0018	1602	2.5022	0.0124
agec65+:days30	0.0045	0.0020	1602	2.2191	0.0266
days30:male	0.0058	0.0016	1602	3.5713	0.0004

* days 30 is defined as taking value 0 until day 30 and increasing from 0 to 110 linearly until 140.

3. Model fixed parameter estimates for comparing kinetics between **omicron-infected and uninfected persons** following the **third** dose. (Standard errors obtained from the model; p-values based on the t-test)

	Value	Std.Error	DF	t-value	p-value
(Intercept)	12.3019	0.0773	936	159.2258	0.0000
male	-0.3782	0.1277	591	-2.9615	0.0032
days30	-0.0193	0.0014	936	-13.8837	0.0000
age65	-0.2308	0.1819	591	-1.2692	0.2049
omic	-0.0569	0.1170	591	-0.4865	0.6268
male:days30	0.0075	0.0024	936	3.1442	0.0017
days30:age65	0.0086	0.0031	936	2.7256	0.0065
age65:omic	0.2137	0.3636	591	0.5879	0.5568
days30:omic	-0.0015	0.0021	936	-0.7261	0.4680
days30:age65:omic	-0.0180	0.0074	936	-2.4289	0.0153

© 2022 Gilboa M et al. *JAMA Network Open*.

eTable 1. Baseline Characteristics of IgG Study Population

	Second dose IgG cohort (n=4868)	Third dose IgG cohort (n=3972)
Number of tests		
1	2237 (46%)	1795 (45%)
2	553 (11%)	802 (20%)
3	297 (6%)	841 (21%)
4	285 (6%)	499 (13%)
5	1496 (31%)	35 (1%)
Gender- male (%)	1310 (27%)	996 (25.1%)
Age-mean+-SD	46.9+-13.7	48.5+-14.1
18-45	2241 (46%)	1704 (43%)
45-65	2072(43%)	1727 (43%)
Over 65	555 (11%)	541 (14%)
BMI	25.5 (+-4.6)	21.3+-10.6
<25	1948 (52%)	1582 (58%)
25-30	1216 (32%)	750 (28%)
>30	446 (12%)	390 (14%)
Immunosuppressed	41 (1%)	25 (1%)
Number of comorbidities		
0	3001 (79%)	2029 (75%)
1	568 (15%)	467 (17%)
>2	239 (6%)	227 (8%)

© 2022 Gilboa M et al. *JAMA Network Open*.

eTable 2. Baseline Characteristics of Neutralizing Antibodies Study Population

	Second dose neutralizing cohort (n=1269)	Third dose neutralizing cohort (n=874)
Number of tests		
1	314 (25%)	311 (36%)
2	179 (14%)	195 (22%)
3	182 (14%)	186 (21%)
4	153 (12%)	150 (17%)
5	306 (24%)	32 (4%)
Gender- male (%)	310 (24%)	253 (29%)
Age-mean	52.7+-14.2	52.5+- 14.0
18-45	398 (31%)	280 (32%)
45-65	527 (42%)	401(34%)
Over 65	344 (27%)	193 (34%)
BMI	26.9+-5.2	23.1+-9
<25	488 (43%)	413 (54%)
25-30	325 (29%)	229 (30%)
>30	326 (29%)	120 (16%)
Immunosuppressed	28 (2%)	8 (1%)
Number of comorbidities		
0	785 (62%)	517 (68%)
1	233 (18%)	151 (20%)
>2	131 (10%)	94 (12%)

© 2022 Gilboa M et al. *JAMA Network Open*.

eTable 3. Baseline Characteristics of T Cell Activation Population

T Cell cohort n=77	
Male	22/77 (29%)
Age	52+-13
<45	27 (35%)
45-60	29 (38%)
>60	21 (27%)
BMI	25.8+-5
Number of comorbidities	
0	50/75 (67%)
1	13/75 (17%)
2+	12/75 (16%)
Immunosuppression	2/75 (3%)

© 2022 Gilboa M et al. *JAMA Network Open*.

eTable 4. Baseline Characteristics of Avidity Study Population

Avidity cohort	n=32
Male	3 (9%)
Age	51.5+-7.6
<45	8 (25%)
45-60	19 (59%)
>60	5 (16%)
BMI	25.0+-7.2
Number of comorbidities	
0	20 (63%)
1	4 (13%)
2+	8 (25%)
Immunosuppression	2 (6%)

© 2022 Gilboa M et al. *JAMA Network Open*.

eTable 5. Baseline Characteristics of Microneutralization Study Population

Microneutralization	1 month<br>n=25	2 months<br>n=25	3 months<br>N=25	4 months<br>N=25
Age	45.2+-15.2	45.3+-14.1	45.3+-12	45.5+-24.1
Male %	6 (24%)	5 (20%)	4(16%)	4 (16%)
Immunosuppression	0/18 (0%)	0/18 (4%)	1/17 (6%)	0/17 (0%)
BMI	24.7+-3.4	25.4+-3.7	27.2+-4.9	28.1+-5.3
Number of comorbidities				
0	14 (78%)	14 (78%)	13 (76%)	8 (47%)
1	2 (11%)	4 (22%)	2 (12%)	6 (35%)
2+	2 (11%)		2 (12%)	3 (18%)

© 2022 Gilboa M et al. *JAMA Network Open*.

eTable 6. Observed Results of Study Population by Time Since Third Dose of Vaccination

Days			**7-28**	29-56	57-84	**85-112**	113-140
IgG		N	296	1721	2296	2049	1730
		GMT	3482	2526	1604	1186	853
		SEM	158	76	44	48	55
Neut		N	340	715	596	531	361
		GMT	3819	3942	3062	2071	1098
		Geometric SD	205	138	148	133	135
Sub-cohort with t cells							
IgG		N	71			71	
		GMT	2342			978	
		Geometric SD	2.3			2.5	
Neut		N	73			73	
		GMT	3722			2134	
		Geometric SD	2.53			3.12	
T cells							
		N	79			79	
		Mean	98			59	
		SEM	5.4			9.3	
		Percent non-responders	7/79 (9%)			16/79 (20%)	
Avidity		N	32		32		
		Mean	97.41		98.04		
		SD	0.103		0.095		
Neutralization		N	25	25	25	25	
	WT (95% CI)		942.3 (585-1518)	1024 (678-1548)	675.6 (443-1029)	248.7 (158-391)	
	Delta (95% CI)		410.1 (266-634)	433.5 (299-629)	310.8 (225-430)	131.8 (88-197)	
	Omicron (GMT+-SEM)		111.4 (75-166)	82.14 (48-140)	55.72 (32-96)	26.14 (16-42)	

© 2022 Gilboa M et al. *JAMA Network Open*.

eTable 7. Mixed Model Analysis of Variables Associated with IgG and Neutralizing Antibody Titers After Second Vaccine Dose

Variable	Peak Titer		Rate of waning		140 days post-vaccine Titer	
	IgG	Neut	IgG	Neut	IgG	Neut
Age group						
<45 yr	Reference	Reference	Reference	Reference	Reference	Reference
45 to >65 yr	0.97 (0.91-1.03)	0.85 (0.74-0.98)	100.1% (100.0-100.1%)	100.3% (100.1-100.6%)	1.07 (1.00-1.14)	1.20 (1.04-1.37)
>= 65 yr	1.00 (0.90-1.10)	0.73 (0.63-0.86)	100.1% (100.0-100.2%)	100.3% (100.0-100.6%)	1.12 (1.01-1.24)	1.03 (0.88-1.21)
Sex						
Female	Reference	Reference	Reference	Reference	Reference	Reference
Male	0.99 (0.90-1.09)	0.73 (0.65-0.84)	100.2% (100.1-100.2%)	100.4% (100.2-100.6%)	1.22 (1.10-1.34)	1.14 (1.00-1.30)

© 2022 Gilboa M et al. *JAMA Network Open*.

eTable 8. Baseline Characteristics of Participants Eventually Infected vs Those Not Infected

	infected	Not Infected
N	1160	1705
Gender- male (%)	225 (19%)	377 (22%)
Age (y)		
Mean	43.8 (95% CI 43.12-44.45)	46.1 (95% CI 45.46-46.74)
18-44	587 (51%)	780 (46%)
45-64	526 (45%)	742 (44%)
≥65	47 (4%)	183 (11%)
Neutralizing antibodies measured	222 (19%)	374 (22%)

© 2022 Gilboa M et al. *JAMA Network Open*.

eTable 9. Computer-Based Questionnaire

	**Question**	**Answer1**	**Answer2**
**1**	What is your date of birth?		
**2**	What is your gender?	Male	Female
**3**	What is your current height in m?		
**4**	What is your current weight in kg?		
**5**	Did you perform an IgG assay before receiving the first dose of the vaccine?	Yes	No
**6**	Do you have high blood pressure disease (systolic blood pressure above 140) treated with medication?	Yes	No
**7**	Do you have dyslipidemia (total cholesterol above 200 or LDL cholesterol above 160) treated with medication?	Yes	No
**9**	Do you have an autoimmune disease treated with medication?	Yes	No
**10**	Do you have diabetes (HbA1C>6.5 or fasting blood sugar>126) treated with medication?	Yes	No
**11**	Do you have heart disease treated with medication?	Yes	No
**12**	Do you have lung diseases such as asthma, COPD, and lung fibrosis treated with medication/s?	Yes	No
**13**	Do you have any coagulation disorder resulting in hemorrhage or thrombosis treated with medication?	Yes	No
**14**	Are you immunosuppressed (organ transplantation, biologic therapy, chemotherapy, steroids, splenectomy, or HIV)?	Yes	No
**15**	Have you ever had a serious allergic reaction (anaphylaxis) that required immediate treatment?	Yes	No
**16**	Do you have a liver disease such as cirrhosis, hepatitis, liver cancer, or metabolic disorder?	Yes	No
**17**	Do you have kidney disease (creatinine>1.2 or GFR<60) treated with medication?	Yes	No
**18**	Are you pregnant (confirmed by a beta HCG blood test and ultrasound fetal heartbeats detection)?	Yes	No

The questionnaire was reviewed and approved by the Institutional review board of the Sheba Medical Center.

IgG=Immunoglobulin G; BMI=Body mass index; Kg=kilogram. M=meter; LDL=low-density lipoproteins; HbA1C=hemoglobin A1C; COPD= chronic obstructive pulmonary disease; HIV=human immunodeficiency; GFR=Glomerular filtration rate.

© 2022 Gilboa M et al. *JAMA Network Open*.

eTable 10. Variable Definitions

Variable	Values	Definitions	Timing
**Outcomes**			
IgG at the peak period	Continuous (S/CO)	SARS-CoV-2 Receptor Binding Domain (RBD) Immunoglobulin G (IgG) assay (Beckman-Coulter, CA, U.S.A.)	During the peak period (days 7-28 after the third vaccination)
NeutAb at the peak period	Continuous (50% titer)	SARS-CoV-2 Pseudo-virus (psSARS-2) Neutralization Assay	During the peak period (days 7-28 after the third vaccination)
IgG in the EoS	Continuous (S/CO)	SARS-CoV-2 Receptor Binding Domain (RBD) Immunoglobulin G (IgG) assay (Beckman-Coulter, CA, U.S.A.)	At the end of the study (day 140 after the third vaccination)
IgG and NeutAb in the EoS	Continuous (50% titer)	SARS-CoV-2 Pseudo-virus (psSARS-2) Neutralization Assay	At the end of the study (day 140 after the third vaccination)
**Variables**			
Sex	Female/male	As defined in SMC' files	Current
Age	Continuous (years)	As defined in SMC' files	At third vaccine dose
BMI	Categorical: <25, 25-29.99, ≥30	BMI was calculated by weight (kg)/(height (m))^2 according to the HCW answer to the questionnaire.	At the second vaccine dose
Blood pressure disease	0/1	According to the HCW answer to the questionnaire: defined as systolic blood pressure above 140 treated with medication	At the second vaccine dose
Dyslipidemia	0/1	According to the HCW answer to the questionnaire: defined as total cholesterol above 200 or LDL cholesterol above 160 treated with medication	At the second vaccine dose
Autoimmune disease	0/1	According to the HCW answer to the questionnaire: defined as a known	At the second vaccine dose

© 2022 Gilboa M et al. *JAMA Network Open*.

		autoimmune disease treated with medication	
Diabetes	0/1	According to the HCW answer to the questionnaire: defined as HbA1C>6.5 or fasting blood sugar>126 treated with medication	At the second vaccine dose
Heart disease	0/1	According to the HCW answer to the questionnaire: defined as known heart disease treated with medication	At the second vaccine dose
Lung disease	0/1	According to the HCW answer to the questionnaire: defined as known lung disease treated with medication	At the second vaccine dose
Coagulation disorder	0/1	According to the HCW answer to the questionnaire: defined as known hemorrhage or thrombosis disease treated with medication	At the second vaccine dose
Immunosuppressed	0/1	According to the HCW answer to the questionnaire: defined as organ transplantation, biologic therapy, chemotherapy, steroids, splenectomy, or HIV	At the second vaccine dose
Allergy	0/1	According to the HCW answer to the questionnaire: defined as a serious allergic reaction (anaphylaxis) that required immediate treatment	During the life
Liver disease	0/1	According to the HCW answer to the questionnaire: defined as cirrhosis, hepatitis, liver cancer, metabolic disorder	At the second vaccine dose
Kidney disease	0/1	According to the HCW answer to the questionnaire: defined as creatinine>1.2 or GFR<60) treated with medication	At the second vaccine dose
Pregnancy	0/1	According to the HCW answer to the questionnaire: defined as confirmed	At the second vaccine dose

© 2022 Gilboa M et al. *JAMA Network Open*.

		pregnancy by a beta HCG blood test and ultrasound fetal heartbeats detection	
Specific comorbidities	Categorical: 0, 1, ≥2	Count of comorbidities that were with significant lower antibodies titers compared to healthy people during the first 5 weeks after the first vaccine dose[3]:<br>• Hypertension<br>• Diabetes<br>• Dyslipidemia<br>• Heart disease<br>• Lung disease<br>• Kidney disease<br>• Liver disease	At the second vaccine dose

Abbreviations: NeutAb= neutralizing antibodies; EoS=end of study; IgG=Immunoglobulin G; S/CO=sample cutoff ratio; SARS-CoV-2=severe acute respiratory syndrome; BMI=Body mass index; Kg=kilogram. M=meter; HCW=health care worker; LDL=low-density lipoproteins; HIV=human immunodeficiency; GFR=Glomerular filtration rate; HbA1C=hemoglobin A1C.

© 2022 Gilboa M et al. *JAMA Network Open*.

A.

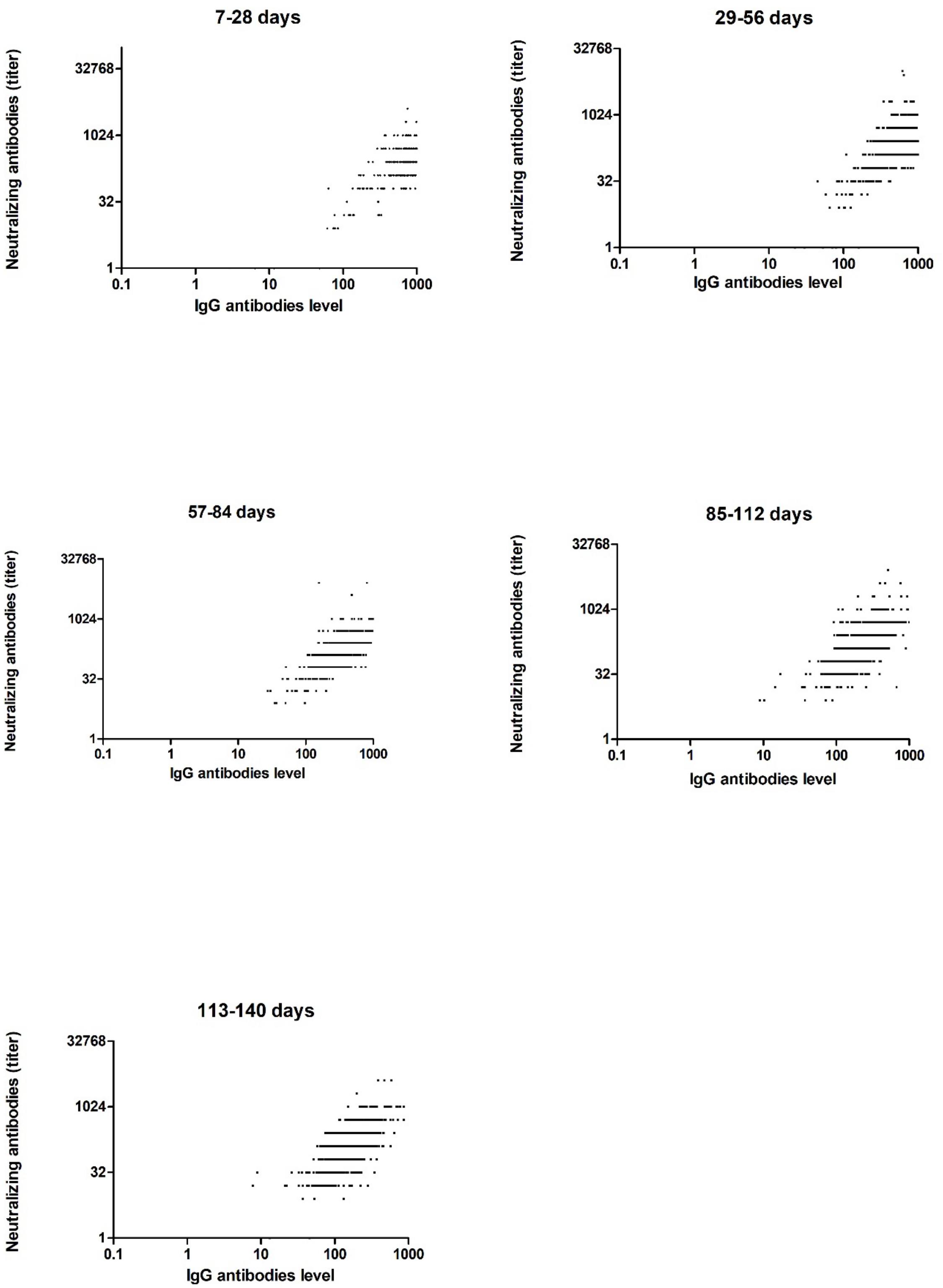

© 2022 Gilboa M et al. *JAMA Network Open*.

B.

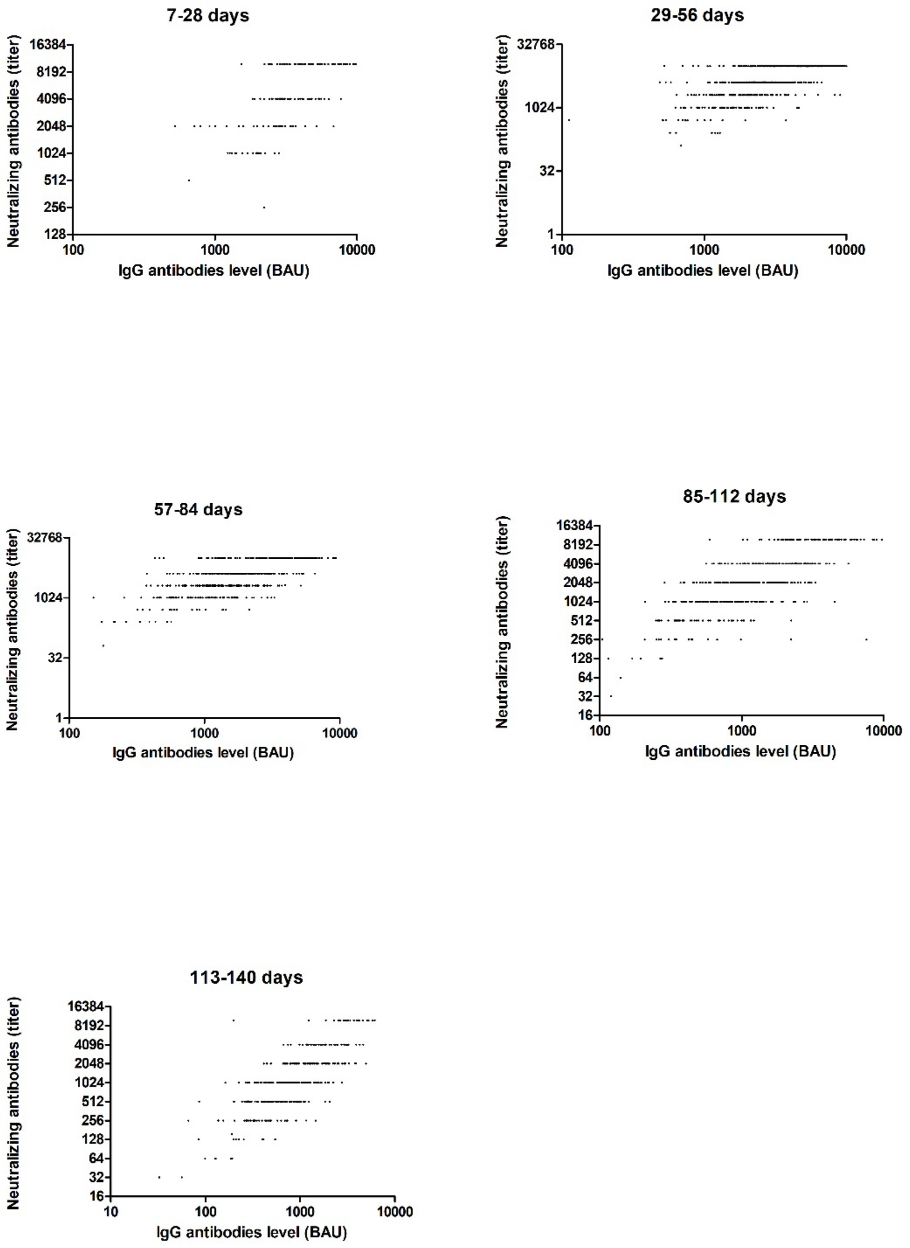

eFigure 1. Correlation Between Neutralizing Antibody Titers and IgG Antibody Titers by Month A. After the second dose of the vaccine, B. after the third dose of vaccine.

© 2022 Gilboa M et al. *JAMA Network Open*.

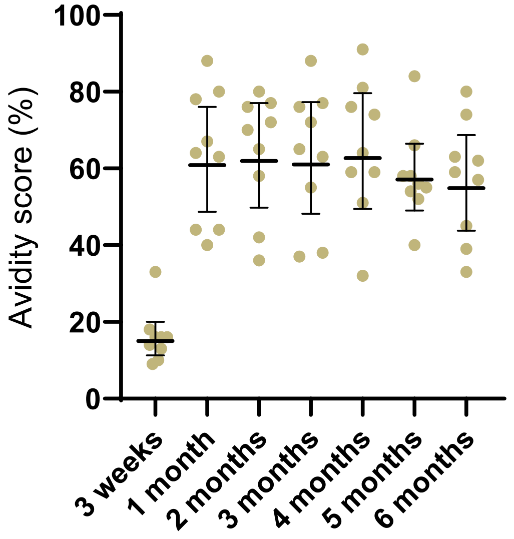

eFigure 2. Antibody Avidity after Second Dose

Antibody avidity from 9 HCW, 3 weeks after the 1st dose and 1-6 months following the 2nd BNT162b2 vaccine dose are presented. The black bars indicate GMT ± 95% CI.

© 2022 Gilboa M et al. *JAMA Network Open*.

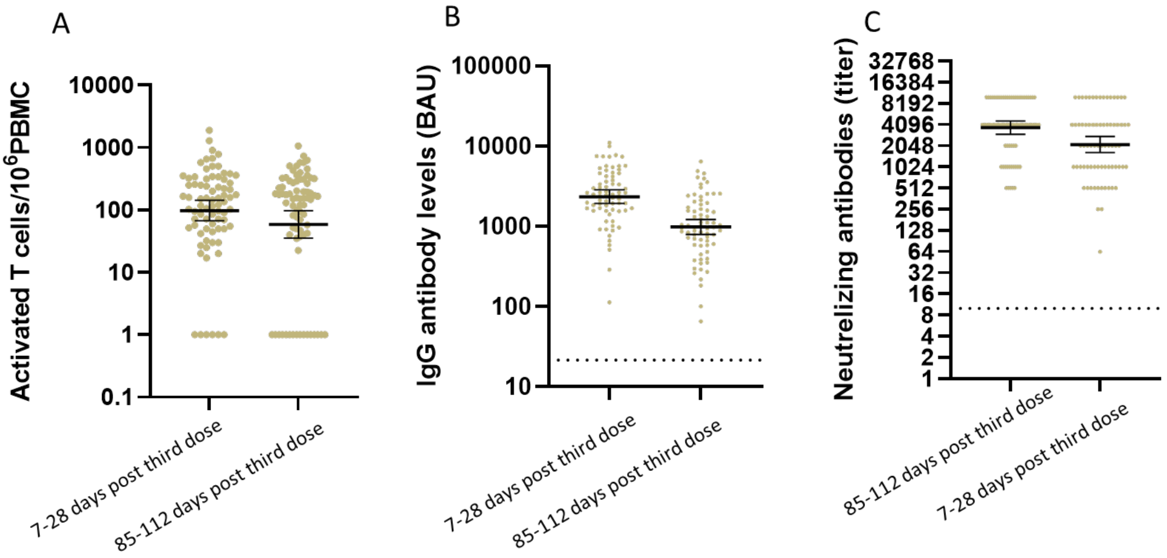

eFigure 3. Immunogenicity of Subgroup of 77 Participants
A. Activated T cells (A), IgG antibodies (B) and neutralizing antibodies (C) 7-28 days and 85-112 days after third dose of vaccination.

© 2022 Gilboa M et al. *JAMA Network Open*.

eReferences

1. Lustig Y, Sapir E, Regev-Yochay G, et al. BNT162b2 COVID-19 vaccine and correlates of humoral immune responses and dynamics: a prospective, single-centre, longitudinal cohort study in health-care workers. Lancet Respir Med 2021;9(9):999–1009.
2. Zuckerman N, Nemet I, Kliker L, et al. The SARS-CoV-2 Lambda variant and its neutralisation efficiency following vaccination with Comirnaty, Israel, April to June 2021. Euro Surveill 2021;26(45).
3. Lustig Y, Zuckerman N, Nemet I, et al. Neutralising capacity against Delta (B.1.617.2) and other variants of concern following Comirnaty (BNT162b2, BioNTech/Pfizer) vaccination in health care workers, Israel. Euro Surveill 2021;26(26).
4. Gilboa M, Mandelboim M, Indenbaum V, et al. Early Immunogenicity and safety of the third dose of BNT162b2 mRNA Covid-19 vaccine among adults older than 60 years; real world experience. J Infect Dis 2021;

© 2022 Gilboa M et al. *JAMA Network Open*.
